# A Single Chain Variant of Factor VIII Fc Fusion Protein Retains Normal In Vivo Efficacy but Exhibits Altered In Vitro Activity

**DOI:** 10.1371/journal.pone.0113600

**Published:** 2014-11-21

**Authors:** Yang Buyue, Tongyao Liu, John D. Kulman, Garabet G. Toby, George D. Kamphaus, Susannah Patarroyo-White, Qi Lu, Thomas J. Reidy, Baisong Mei, Haiyan Jiang, Glenn F. Pierce, Jurg M. Sommer, Robert T. Peters

**Affiliations:** Hematology Research, Biogen Idec, Cambridge, Massachusetts, United States of America; Emory University School of Medicine, United States of America

## Abstract

Recombinant factor VIII Fc (rFVIIIFc) is a fusion protein consisting of a single B-domain-deleted (BDD) FVIII linked recombinantly to the Fc domain of human IgG1 to extend half-life. To determine if rFVIIIFc could be further improved by maintaining the heavy and light chains within a contiguous single chain (SC), we evaluated the activity and function of SC rFVIIIFc, an isoform that is not processed at residue R1648. SC rFVIIIFc showed equivalent activity in a chromogenic assay compared to rFVIIIFc, but approximately 40% activity by the one-stage clotting assay in the presence of von Willebrand Factor (VWF), with full activity in the absence of VWF. Moreover, SC rFVIIIFc demonstrated markedly delayed thrombin-mediated release from VWF, but an activity similar to that of rFVIIIFc upon activation in FXa generation assays. Therefore, the apparent reduction in specific activity in the aPTT assay appears to be primarily due to delayed release of FVIII from VWF. To assess whether stability and activity of SC rFVIIIFc were affected in vivo, a tail vein transection model in Hemophilia A mice was utilized. The results demonstrated similar pharmacokinetic profiles and comparable efficacy for SC rFVIIIFc and rFVIIIFc. Thus, while the single chain configuration did not promote enhanced half-life, it reduced the rate of release of FVIII from VWF required for activation. This impaired release may underlie the observed reduction in the one-stage clotting assay, but does not appear to affect the physiological activity of SC rFVIIIFc.

## Introduction

Hemophilia A is an X-linked bleeding disorder caused by deficiency of factor VIII (FVIII) activity [1],[2]. Although prophylaxis is considered the standard of the care [1],[2], compliance with the regimen is hampered by the short half-life (∼12 hours) of FVIII that requires dosing every other day or three times per week by intravenous injection to maintain a minimum plasma level of 1% of normal coagulation factor activity [3],[4]. A novel recombinant factor VIII Fc fusion protein (rFVIIIFc) with prolonged plasma half-life (1.5–1.7 fold) was developed to reduce prophylactic injection frequency [5],[6]. In a phase 3 open-label, multicenter, partially randomized study, rFVIIIFc resulted in low bleeding rates when dosed 1–2 times per week in patients with severe hemophilia A [7].

rFVIIIFc consists of a single molecule of B-domain deleted (BDD) rFVIII covalently linked to the dimeric human Fc region from IgG_1_ with no intervening linker sequence. rFVIIIFc is produced in stably transfected human embryonic kidney 293 cells (HEK293) cells, with a molecular weight of approximately 220 kDa. Direct fusion of the Fc portion of the ubiquitous human immunoglobulin G extends the half-life of proteins by binding of Fc to the neonatal Fc receptor (FcRn), which delays lysosomal degradation by cycling immunoglobulins and Fc fusion proteins back into circulation, and thus leverages the same natural pathway responsible for the long plasma half-life of IgG [8],[9]. rFVIIIFc is expressed as two polypeptide chains, one chain consisting of the Fc domain (hinge, CH_2_ and CH_3_) of human IgG_1_, the other chain consisting of BDD rFVIII fused to the same Fc region. The B domain deletion is created by fusing Ser 743 (S743) to Gln 1638 (Q1638) with respect to the full length FVIII sequence resulting in a 14 amino acid sequence from the original B domain [10].

**Figure 1:**
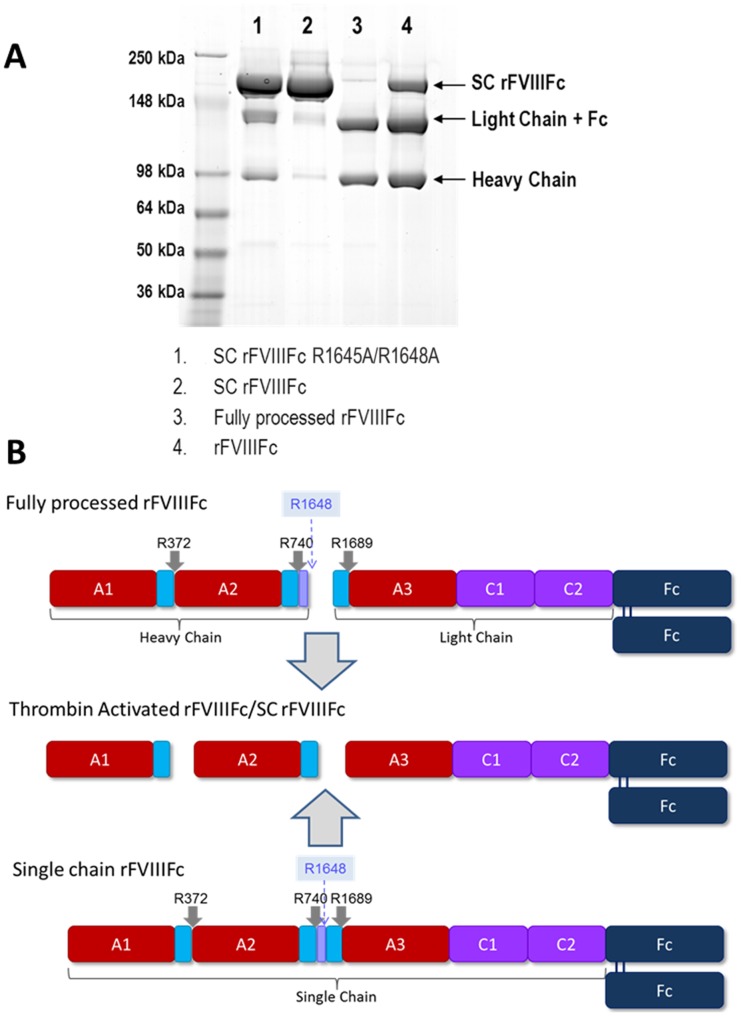
Non-Reducing SDS-PAGE analysis and schematic of FVIII variants. (A) SC rFVIIIFc R1645A/R1648A, SC rFVIIIFc, fully processed rFVIIIFc and rFVIIIFc [10] were compared by non-reducing SDS-PAGE. (B) Schematic presentation of SC rFVIIIFc, fully processed rFVIIIFc, and the common thrombin-activated form.

During secretion, the majority of BDD rFVIIIFc is processed intracellularly by proteolytic cleavage after Arg1648 (numbering based on full length FVIII sequence) to generate an approximately 90 kDa heavy chain (HC) and an approximately 130 kDa light chain (LC)-Fc fusion (Figure 1). Upon activation in plasma, BDD rFVIII is cleaved by thrombin after three arginine residues, at positions 372, 740 and 1689, to generate rFVIIIa consisting of the 50 kDa A1, 43 kDa A2, and 73 kDa A3-C1-C2 chains [11],[12]. These cleavages also release the a3 acidic domain from the N-terminus of the LC, which is required for Von Willebrand factor (VWF) binding to FVIII, as well as the remaining 14 amino acids of the B domain [13]. It has been reported that in a number of recombinant factor FVIII molecules [14]–[17], the cleavage at R1648 does not occur for a fraction of the secreted BDD rFVIII product, leading to the generation of non-processed single chain rFVIII isoform. Also, there are recent reports on different isoforms of SC rFVIII designed to improve in vivo activity and prolong half-life [18]–[20].

To understand the function and activity of SC rFVIIIFc, we purified this isoform and assessed its activity employing a variety of *in vitro* assays including one-stage clotting assay (aPTT-based), two-stage chromogenic assay, thrombin generation assay and enzymatic activity and intermolecular affinity assays using synthetic membrane surfaces. The affinity of SC rFVIIIFc to human VWF and the thrombin-mediated release from VWF were analyzed with surface plasmon resonance (SPR). Finally, *in vivo* studies using a HemA mouse model were performed to investigate the pharmacokinetics and efficacy. While this study focuses on SC rFVIIIFc, the results may offer insights into the biochemical and physiological characteristics of other single chain rFVIII molecules.

## Material and Methods

### Cloning, expression, and purification of rFVIIIFc, SC rFVIIIFc and fully processed rFVIIIFc

rFVIIIFc (Figure 1A, lane 4) was produced as previously described [5],[10]. Two forms of a single chain rFVIIIFc were generated: one form, SC rFVIIIFc, was purified from rFVIIIFc (Figure 1A, lane 2) and the other form, SC rFVIIIFc R1645A/R1648A, was created through introduction of mutations into the intracellular processing site (R1645A/R1648A) of the rFVIIIFc construct (Figure 1A, lane 1). SC rFVIIIFc was purified from rFVIIIFc as described in the Supporting Information. SC rFVIIIFc R1645A/R1648A was produced in a similar manner to rFVIIIFc [10] after establishing a stable cell line with a rFVIIIFc construct in which the processing site was mutated using standard molecular biology techniques. Fully processed rFVIIIFc (Figure 1A, lane 3) was produced by co-transfection of rFVIIIFc with human PC5, a member of the proprotein convertase subtilisin/kexin (PCSK) type proteases, as described [10]. The proteins were analyzed by non-reducing sodium dodecyl sulfate polyacrylamide gel electrophoresis (SDS-PAGE) and the mass concentrations determined by measuring UV absorbance at 280 nm.

### FVIII comparators

Two commercially available antihemophilic factors (recombinant) were utilized in these studies: rBDD FVIII ReFacto and Xyntha (Wyeth Pharmaceuticals, Philadelphia, PA, USA) were purchased and reconstituted according to manufacturers' guidelines.

### Thrombin and lysyl endopeptidase peptide mapping

Procedures were carried out as previously described [10]. rFVIII and rFVIIIFc samples were fully digested with thrombin, reduced, then analyzed by either reversed phase high-performance liquid chromatography with ultraviolet detection (RP-HPLC/UV) or reversed phase high-performance liquid chromatography/mass spectrometry (RP-HPLC/MS). Peptide sequence was confirmed with lysyl endopeptidase (LysC) peptide mapping and analyzed by RP-HPLC/MS.

### Automated aPTT (one-stage clotting assay) and chromogenic assay

FVIII samples were analyzed by one-stage clotting assay (Actin FSL reagent, Siemens, Malvern, PA) or by chromogenic assay (Siemens Healthcare) on a Sysmex CA 1500 instrument using Multiple Dilution Analysis and activity calculated relative 8th WHO International Standard Factor VIII Concentrate (NIBSC code 07/350). Each sample was analyzed by the one-stage clotting assay and two-stage chromogenic assays simultaneously with 3 dilutions in triplicate in each run. Factor VIII-deficient plasma was obtained from HRF Inc. (Raleigh, NC) and FVIII and VWF-immuno depleted plasma was purchased from Stago US (Parsippany, NJ). The dose formulation analysis in the HemA mouse efficacy study was done with a FVIII ELISA assay using a monoclonal anti-FVIII antibody GMA8016 (Green Mountain Antibodies, Burlington, VT).

### Thrombin generation assay

Thrombin activity was determined by the calibrated automated thrombogram (CAT) method described by Hemker et al. using the standard assay protocol and reagents from Thrombinoscope (Stago, Parsippany, NJ) [21]. Final concentrations of reagents were 1 pM tissue factor and 4 µM phospholipids for assay wells, or 630 nM thrombin calibrator for calibration wells.

### Surface plasmon resonance (SPR) analysis of affinity for VWF

The affinities of rFVIIIFc and SC rFVIIIFc for human plasma-derived VWF were determined with a Biacore T100 SPR instrument (GE Healthcare, Piscataway, NJ) as described previously [10] and in detail in the Supporting Information.

### SPR analysis of thrombin-mediated release of activated FVIII variants from VWF

Thrombin-mediated FVIII release assays were performed with a Biacore T100 instrument, and consisted of four steps, as described in detail in the Supporting Information. Briefly, human plasma-derived VWF was immobilized on flow cells, and FVIII variants were infused over this surface to achieve normalized capture levels. Human α-thrombin at different concentrations was then applied, and the resulting rates of release for different activated FVIII variants were determined.

### Activity in Xase complex by Factor Xa generation assay

FIXa, FX, FXa and human α-thrombin were purchased from Haematologic Technologies (Essex Junction, Vermont). Hirudin and FXa substrates Pefachrome 6034 were obtained from Centerchem (Norwalk, CT). The phospholipids (25% phosphatidylserine and 75% phosphatidylcholine) were purchased from Avanti Polar Lipids (Alabaster, AL) and prepared by extrusion through a 100-nm polycarbonate filter to get homogenous mixture [22]. Methods and analysis for determining activity in Xase complex were performed as described [10],[23],[24]. In general, FVIII was first activated with α-thrombin for 5 minutes, then stopped with hirudin and mixed with FIXa in the presence of Ca^2+^ and the phospholipids. FVIIIa and FIXa interacted to form an active Xase complex that mediated the conversion of FX into FXa through proteolytic processing. In turn, FXa cleaved an FXa-specific chromogenic substrate and the amount of cleaved substrate in a solution wass indicative of the amount of FXa generated. This was quantified by measuring the absorbance of the solution at 405 nm and the kinetic parameters determined for each independent run. These parameters were then averaged and expressed as mean ± standard deviation.

### Pharmacokinetic (PK) Studies in HemA mice

The PK of purified SC rFVIIIFc and rFVIIIFc was evaluated in HemA mice as previous described [5] after a single intravenous dose of 250 IU/kg. Blood was collected from the vena cava in one-tenth volume of 4% sodium citrate at 15 minutes, and 8, 24, 48, 72 and 96 hrs post-dosing (4 mice/time point/treatment). Plasma was frozen and stored at −80°C until analyzed for FVIII activity using a FVIII chromogenic assay on Siemens Sysmex CA1500. The pharmacokinetic parameters were estimated by non-compartmental modeling using WinNonLin version 5.2 (Pharsight, Mountain View, CA).

### Efficacy evaluation in tail vein transection (TVT) bleeding model in HemA mice

All efficacy studies were performed blinded. The tail vein transection (TVT) bleeding model was conducted as described previously [25] except that HemA mice received a single intravenous administration of 0.46 µg/kg, 1.38 µg/kg or 4.6 µg/kg of SC rFVIIIFc or rFVIIIFc at 48 hours prior to the transection of a lateral tail vein. The log-rank (Mantel-Cox) test was used for statistical analysis.

### Ethics Statement

This study was carried out in strict accordance with the recommendations in the Guide for the Care and Use of Laboratory Animals of the National Institutes of Health. All study protocols were reviewed and approved by the Institutional Animal Care and Use Committee (IACUC) of Biogen Idec (Permit Number: 01-10). All surgery was performed under anesthesia, and all efforts were made to minimize suffering [25]. More specifically, at the desired time point, the mice were anesthetized with a Ketamine/Dexmedetomidine/Buprenex cocktail. This cocktail provided a Ketamine dose of 50 mg/kg, a Dexmedetomidine dose of 0.5 mg/kg, a Buprenex dose of 0.1 mg/kg (when injected at 5 ml/kg of body weight intraperitoneally). For the mouse that could not reach adequate anesthetic depth, another 50 ul of 10 mg/ml of Ketamine, approximately 20 mg/kg (mix 1 ml of 100 mg/ml of Ketamine solution with 9 ml of sterile saline) were injected. After tail vein transection, the mouse was returned to its individual cage with white paper bedding, placed on top of a heating pad. In the following 11 hours and then overnight at 24 hours, the study animals were monitored hourly and euthanized immediately with lethal dose of CO_2_ when they reached moribund state, which is defined as being recumbent and unresponsive to external stimuli [25]. At the last time point before going overnight, the remaining mice received 0.1 mg/kg Buprenex for pain relief. All mice were euthanized at 24 hours after tail vein transection.

## Results

### Purification and characterization of the SC rFVIIIFc isoforms

SC rFVIIIFc differs from rFVIIIFc solely by the absence of single peptide bond between R1648 and E1649. Therefore, a method was developed to separate these nearly identical molecules. Chelation of the divalent cations of FVIII was used to disrupt the HC:LC interactions, creating three distinct molecules (free HC, LC-Fc, and SC rFVIIIFc) that were then separated through a combination of anion exchange and an affinity chromatography steps, and the divalent cations were then reintroduced in specific concentrations to allow for the re-establishment of the HC:LC interactions. Size exclusion chromatography was then utilized to remove any aggregated species formed.

SC rFVIIIFc was analyzed by non-reducing sodium dodecyl sulfate polyacrylamide gel electrophoresis (SDS-PAGE) (Figure 1A, lane 2) and the protein was detected at approximately 220 kDa, consistent with the predicted molecular weight for SC rFVIIIFc, and only trace amounts of processed rFVIIIFc (LC-Fc and HC) were detected. The rFVIIIFc starting material was also analyzed, and shown to contain a majority of processed rFVIIIFc (LC-Fc and HC) as well as SC rFVIIIFc (Figure 1A, lane 4) as previously reported [5],[10]. A recombinantly engineered single chain FVIIIFc (SC rFVIIIFc R1645A/R1648A, Figure 1A, lane 1) was produced to confirm results obtained with SC rFVIIIFc by introducing mutations into the intracellular processing site (R1645A/R1648A) in the rFVIIIFc construct. A fully processed rFVIIIFc protein (Figure 1A, lane 3) was also produced by co-transfection of PC5, as previously described [10].

All species were analyzed by peptide mapping with LysC digests followed by UV and mass spectrometric detection. SC rFVIIIFc exhibited a peptide map identical to rFVIIIFc, with identical primary sequence and post translational modifications, with the exception that only peptides with an intact processing site were detected, with no evidence of the peptides cleaved at the processing site (data not shown). This observation was confirmed by thrombin digestion followed by LC-MS (TOF) analysis (Figure S1 in File S1). These analyses also confirmed the absence of other truncated products in the SC rFVIIIFc (e.g. HC truncations due to cleavage after E720 or Y729, or LC truncations before D1658) that were found in other rFVIII products, as well as rFVIIIFc (Figure S1, S2; Table S1 in File S1). The SC rFVIIIFc R1645A/R1648A was found to have a similar peptide map to the purified SC rFVIIIFc, with the main difference that the R1645A/R1648A mutations were confirmed and no cleavage at this mutant site was found (Figure S1 in File S1). Also, the SC rFVIIIFc R1645A/R1648A contained low levels of the truncated forms at E720, Y729 and D1658 (Figure S1, S2; Table S1 in File S1), accounting for the apparent LC-Fc and HC observed by SDS-PAGE (Figure 1, lane 1). The SC rFVIIIFc R1645A/R1648A was also found to have slight variations in glycosylation patterns in both the FVIII and Fc regions by both the LysC and thrombin peptide mapping (data not shown).

### Activity comparison in chromogenic and one-stage assays

SC rFVIIIFc, fully processed rFVIIIFc, or rFVIIIFc were diluted in either human congenital factor VIII-deficient plasma which contained VWF or FVIII immuno-depleted plasma lacking VWF (<1% VWF). All three forms of rFVIIIFc demonstrated comparable activity by the chromogenic assay regardless of VWF presence in plasma (Table 1). In the aPTT assay, the fully processed rFVIIIFc and rFVIIIFc demonstrated comparable specific activity in the presence or absence of VWF, while SC rFVIIIFc had approximately 40% specific activity when tested in congenital deficient plasma containing normal levels of VWF.

**Table 1:** Specific activity of SC rFVIIIFc compared to fully processed rFVIIIFc and rFVIIIFc by chromogenic and one-stage (aPTT) assays (n = 3).

Matrix	Sample	Chromogenic Specific Activity (IU/mg): Mean	% CV	Coagulation (aPTT) Specific Activity (IU/mg): Mean	%CV
Congenital FVIII-deficient plasma	SC rFVIIIFc	8194	2.7	3108	6.6
	Fully processed rFVIIIFc	9577	8.3	8683	3.6
	rFVIIIFc	9066	2.5	8210	5.9
FVIII/VWF-depleted plasma	SC rFVIIIFc	9498	4.7	13572	2.4
	Fully processed rFVIIIFc	9569	4.5	15170	10.4
	rFVIIIFc	10801	8.9	15621	6.5
FVIII/VWF-depleted plasma supplemented with human VWF	SC rFVIIIFc	8984	4.6	3133	4.9
	Fully processed rFVIIIFc	8275	8.2	8495	4.0
	rFVIIIFc	9982	4.3	7742	7.4

When the FVIII/VWF-depleted plasma was supplemented with 10.5 nM VWF, the clotting activity of SC rFVIIIFc measured by aPTT returned to the reduced activity observed in human congenital FVIII-deficient plasma (Table 1). The chromogenic activity of SC rFVIIIFc in this condition remained equivalent to the other two comparators. rFVIIIFc and fully processed rFVIIIFc demonstrated comparable activity in both assays in FVIII/VWF-depleted plasma with VWF supplementation.

### Moderately reduced thrombin generation profile of SC rFVIIIFc relative to rFVIIIFc, fully processed rFVIIIFc and WHO FVIII concentrate standard in the presence of VWF

**Figure 2:**
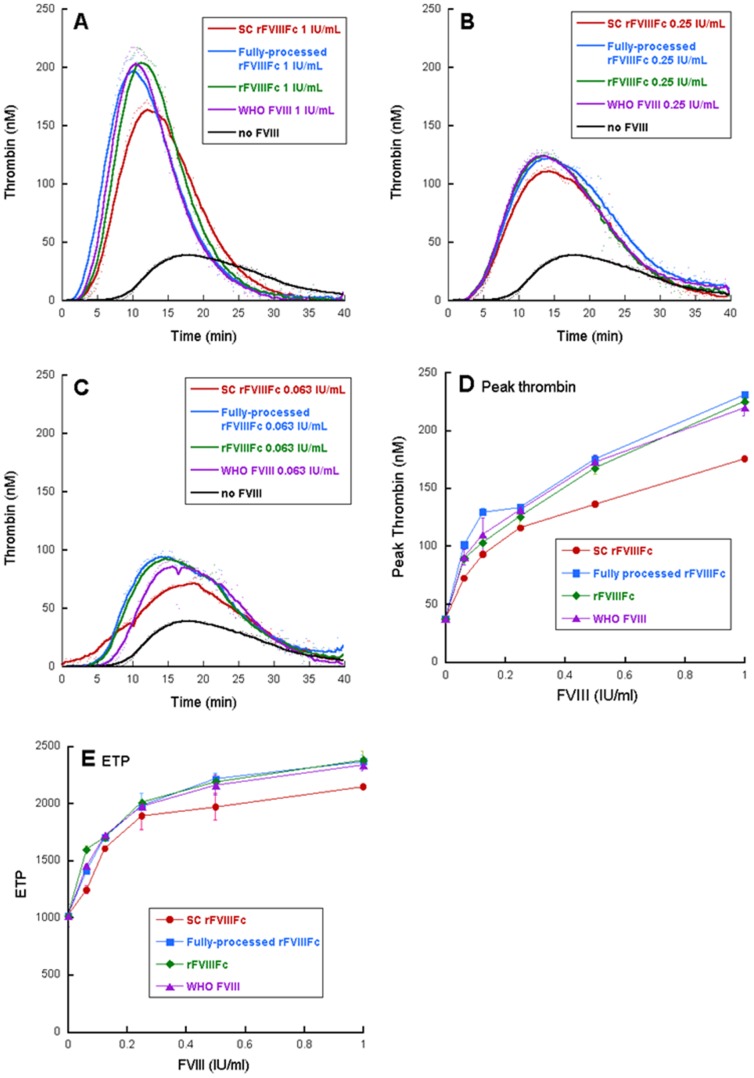
Thrombin generation profile of FVIII variants. Activity determination was based on equal chromogenic activity. Representative curves with selected concentrations are shown in (A) 1 IU/mL (B) 0.25 IU/mL (C) 0.0625 IU/mL. Select parameters are shown in (D) as peak thrombin and (E) as ETP.

To evaluate the ability of FVIII isoforms to support thrombin generation, each sample was added to human congenital FVIII-deficient plasma at 0, 0.063, 0.125, 0.25, 0.5 and 1 IU/mL based on the activity measured by the automated chromogenic assay (Figure 2). Thrombin generation was triggered with 1 pM tissue factor in the presence of 4 µM phospholipids. When no factor VIII is present in the plasma, baseline activation of thrombin was detected with a peak thrombin generation of 40 nM. Generally, increasing concentrations of factor VIII supported enhanced peak height, area under the curve (ETP) and shortened time to peak. SC rFVIIIFc demonstrated moderately reduced peak thrombin height and ETP, while rFVIIIFc, fully processed rFVIIIFc and the WHO FVIII concentrate standard generated similar thrombin generation profiles in all concentrations (Figure 2).

### Comparable Activity in Xase complex

**Figure 3:**
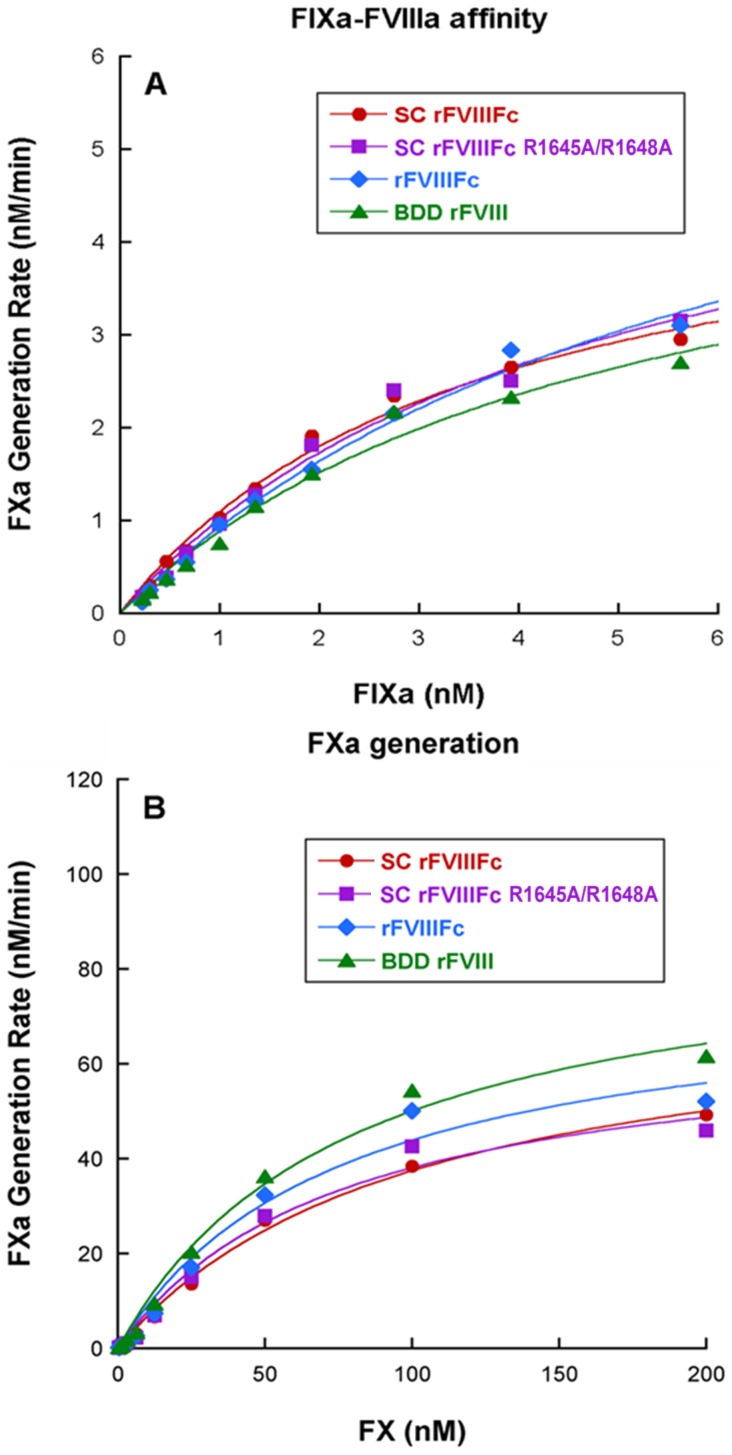
Activity within FXa generation assays. (A) Affinity of FIXa to different FVIII molecules on synthetic phospholipids surface (B) FXa generation rates for Xase Complexes formed with different FVIII molecules. A representative curve is shown. All results are listed in Table 2.

To evaluate the affinity of SC rFVIIIFc to FIXa and the ability to generate FXa, the rates of FXa generation by the Xase complex (FVIIIa-FIXa complex) were assessed in a FXa generation assay where the formation of FXa was monitored by the cleavage of a FXa chromogenic substrate at 405 nm. Thrombin-activated SC rFVIIIFc returned a *K*_D_ value of 4.1±0.6 nM, comparable to activated rFVIIIFc (5.5±1.1 nM) and BDD rFVIIIa (ReFacto, Wyeth Inc.) (4.5±0.6 nM). The *V*_max_ values for these molecules were also comparable (Figure 3, Table 2). We analyzed the activity of the Xase complex formed between the different FVIIIa molecules and FIXa by determining the affinity (*K*_M_) of the Xase complex to the substrate FX. The average *K*_M_ and *V*_max_ values from multiple runs showed that the Xase complex formed by thrombin activated SC rFVIIIFc, rFVIIIFc and BDD rFVIII molecules exhibited similar affinities towards FX and that the *V*_max_ values were comparable.

**Table 2:** Comparison of FIXa binding to FVIIIa within Xase complex and FX interaction with Xase complex assembled with FVIIIa on synthetic phospholipids (n = 6, Mean ± SD) [10].

	SC rFVIIIFc	SC rFVIIIFc R1645A/R1648A	rFVIIIFc [10]	BDD rFVIII [10]
Apparent *K*_D_ (FIXa-FVIIIa affinity) (nM)	4.1±0.6	3.8±0.5	5.5±1.1	4.5±0.6
*V*_max_ (FIXa-FVIIIa affinity) (nM/min)	5.4±0.7	5.9±0.4	6.9±0.7	5.7±1.3
*K*_M_ (FXa generation) (nM)	85.9±13.6	93.6±12.7	90.9±11.9	69.2±11.8
*V*_max_ (FXa generation) (nM/min)	77.8±17.3	68.8±10.8	86.4±10.7	86.5±12.2

### Comparable Affinities of FVIII isoforms for VWF

The affinities of SC rFVIIIFc and rFVIIIFc for human VWF were determined by surface plasmon resonance (SPR). Both SC rFVIIIFc and rFVIIIFc exhibited high affinity for VWF with similar *K*_D_ values (3.4±0.1×10^−10^ M and 3.1±0.1×10^−10^ M, respectively; Table 3 and Figure S3 in File S1).

**Table 3:** Affinities of SC rFVIIIFc and rFVIIIFc for VWF by SPR (n = 6, Mean ± SD) [10].

FVIII	*k*_a_, M^−1^s^−1^	*k*_d_, s^−1^	*K*_D_, M
SC rFVIIIFc	2.7±0.1×10^6^	8.4±0.4×10^−4^	3.1±0.1×10^−10^
rFVIIIFc [10]	2.6±0.4×10^6^	8.9±1.3×10^−4^	3.4±0.1×10^−10^

### Delayed thrombin-mediated release of SC rFVIIIFc from VWF

**Figure 4:**
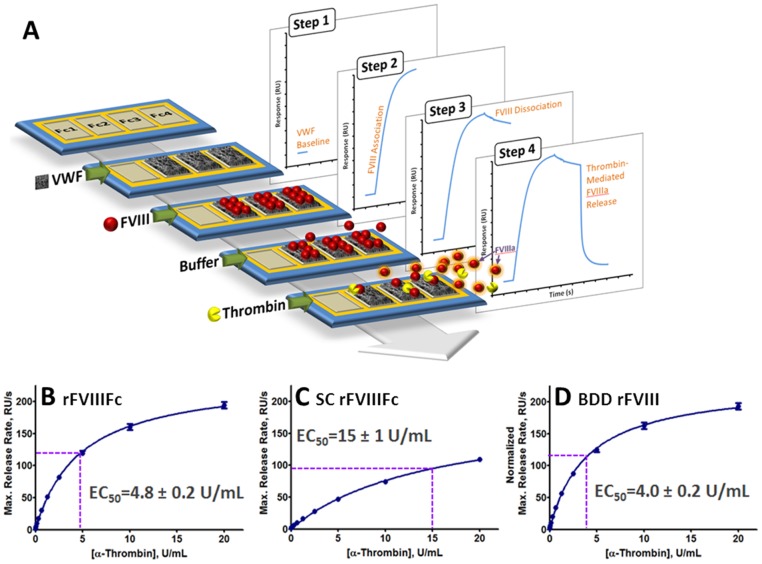
Thrombin-mediated release of activated FVIII variants from VWF. (A) Schematic of the optical biosensor method developed to evaluate the dependence of VWF release rates on thrombin concentration. A detailed description is provided in *Materials and Methods*. (B–D) Determination of thrombin concentrations corresponding to half-maximal release rates (EC_50_) for (B) rFVIIIFc, (C) SC rFVIIIFc, and (D) BDD rFVIII. For BDD rFVIII, ordinate values were normalized on a molar basis to account for the molecular weight difference between rFVIIIFc and SC rFVIIIFc (220 kDa), and BDD rFVIII (170 kDa).

The thrombin-mediated release of activated FVIII isoforms from VWF was evaluated at 25°C and 37°C by using a novel SPR-based optical biosensor method (Figure 4A). At 37°C, the thrombin half-maximal effective concentration (EC_50_) for release from VWF was 15±1 U/mL for SC rFVIIIFc, 4.8±0.2 U/mL for rFVIIIFc and 4.0±0.2 U/mL of BDD rFVIII (Figure 4B–D and Figure S5 in File S1). Similar relative rates were observed at 25°C, with thrombin EC_50_ values for release from VWF of 12±1 U/mL for SC rFVIIIFc, 3.9±0.3 U/mL for rFVIIIFc, and 3.3±0.3 U/mL for BDD rFVIII (Figure S4 in File S1).

### Equivalent PK of SC rFVIIIFc and rFVIIIFc in HemA mice

**Figure 5:**
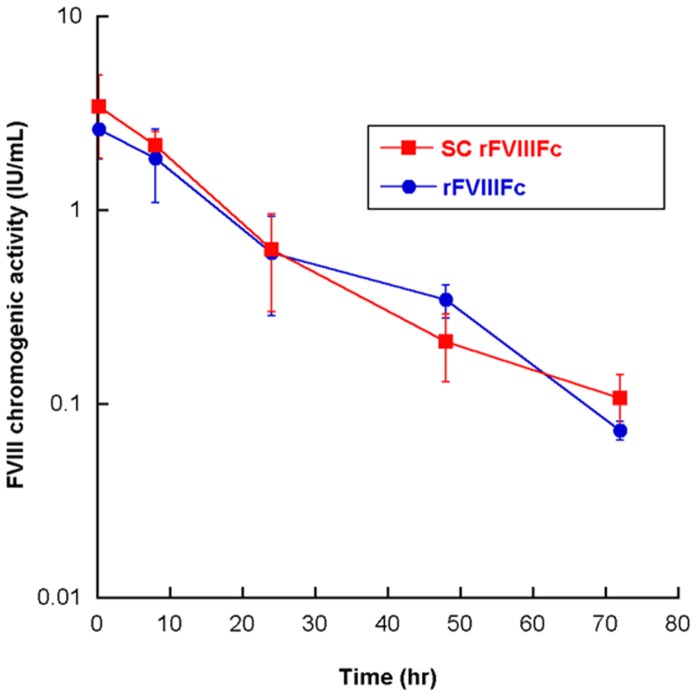
PK profiles of SC rFVIIIFc and rFVIIIFc in HemA mice (single dose 250 IU/kg). Results shown are mean ± SD from 4 mice per treatment at each time point. The PK parameter estimates are summarized in Table 4.

**Figure 6:**
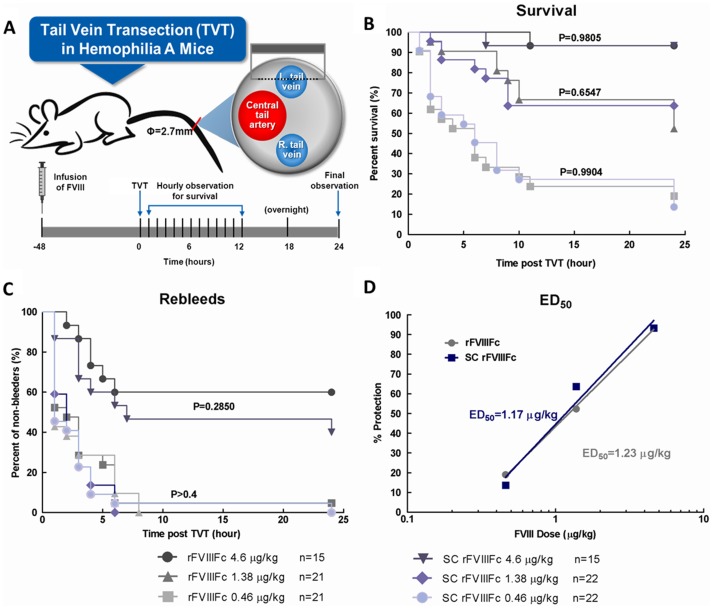
SC rFVIIIFc and the rFVIIIFc demonstrated equivalent in vivo efficacy in a HemA mouse bleeding model. Tail-bleeding of HemA mice was induced via transection of one of the lateral veins at 48 hr post i.v. dosing of SC rFVIIIFc or rFVIIIFc (0.46, 1.38 and 4.6 µg/kg). Mice were monitored for survival and re-bleeding for 24 hr post injury. (A) Schematic experimental design of TVT model. (B) 24 hr post TVT survival curves (C) 24 hr post TVT re-bleeding curves from experimental animals. (D) The linear regression curve of the percentage of protection (survival rate) versus the log (base 10) of the dose was plotted. The ED_50_ was extrapolated from the curves.

The PK of SC rFVIIIFc and rFVIIIFc (Figure 5) was studied in HemA mice at a single dose of 250 IU/kg. The PK parameters (Table 4) were determined by the chromogenic measurement of the human FVIII activity in mouse plasma. The half-life of SC rFVIIIFc is 13.8 hours (Figure 5 & Table 4), similar to that of rFVIIIFc activity (Figure 6B) and previously described [5]. All other parameters are comparable between SC rFVIIIFc and rFVIIIFc.

**Table 4:** Summary of PK parameters for SC rFVIIIFc and rFVIIIFc.

PK parameters	unit	SC rFVIIIFc	rFVIIIFc
t_1/2_	hr	13.8	14.5
C_max_	µg/ml	0.4	0.3
AUC	hr*µg/ml	7.2	6.7
Vss	ml/kg	83.2	94.6
Cl	ml/hr/kg	4.2	4.5
MRT	hr	16.7	19.3

### SC rFVIIIFc has comparable activity to rFVIIIFc in the tail vein transection (TVT) bleeding model in HemA mice

To compare in vivo efficacy of SC rFVIIIFc and rFVIIIFc, male HemA mice were treated with the escalating doses (0.46–4.6 µg/kg) of either SC rFVIIIFc or rFVIIIFc 48 hours prior to TVT injury. The rates of re-bleed and survival were recorded hourly in the first 12 hours and then 24-hour post TVT, with moribund animals euthanized at time of observation (Figure 6A). Both SC rFVIIIFc and rFVIIIFc were fully active in this model resulting in nearly 100% survival at 4.6 µg/kg. Furthermore, both SC rFVIIIFc and rFVIIIFc had demonstrated highly comparable dose responses in the survival curve (Figure 6B) and rebleed rate (Figure 6C). The effective doses of SC rFVIIIFc and rFVIIIFc to achieve 50% survival (ED_50_) were comparable at 1.17 µg/kg and 1.23 µg/kg respectively, similar to rFVIIIFc results reported previously [5]. No evidence of enhanced in vivo stabilized activity of SC rFVIIIFc was observed.

## Discussion

Recombinant factor VIII Fc has shown comparable specific activity in vitro and efficacy in vivo relative to rFVIII, with approximately 1.6-fold increased plasma half-life in hemophilia A patients [5]–[7] [10]. Here we characterized a single chain isoform of rFVIIIFc to determine if it provides comparable or improved activity relative to rFVIIIFc. Our results indicate that SC rFVIIIFc is fully active and functional despite its reduced activity under certain conditions in one-stage clotting assay and thrombin generation assay. This conclusion is supported by several lines of evidence generated in the chromogenic FVIII activity assay, in detailed biochemical characterizations of the binding affinity to FIXa, and in the ability to activate FX in the Xase complex. Furthermore, SC rFVIIIFc exhibits comparable PK and in vivo efficacy to rFVIIIFc, as demonstrated by results from the comparison with rFVIIIFc in a HemA mouse tail vein transection model, which showed superior in vivo efficacy compared to rFVIII due to the prolonged half-life conferred by the Fc moiety [5].

We first obtained a high-purity SC rFVIIIFc using a novel purification strategy and showed that this isoform had equivalent primary sequence and post translational modification to rFVIIIFc (Figures S1 and S2, Table S1 in File S1). When analyzed for activity in either congenital FVIII-deficient plasma with normal VWF or in FVIII/VWF-depleted plasma, SC rFVIIIFc demonstrated comparable specific activity to rFVIIIFc in the two-stage chromogenic assay (Table 1). In the one-stage clotting assay (aPTT), SC rFVIIIFc demonstrated 40% activity when the FVIII-deficient plasma had normal VWF level but equivalent activity in FVIII/VWF-depleted plasma (Table 1), suggesting the potential role of VWF in the delayed activation of SC rFVIIIFc. This observation was further confirmed by addition of human VWF back to the FVIII/VWF-depleted plasma (Table 1), which reduced the coagulant activity of SC rFVIIIFc to the same level as in congenital FVIII-deficient plasma. Interestingly, although rFVIIIFc contains a minor amount of SC rFVIIIFc (Figure 1 lane 4) [10], the activity of rFVIIIFc in chromogenic or aPTT assays has been consistently comparable to the fully processed rFVIIIFc under all conditions tested in these studies (Table 1).

An alternate form of SC rFVIIIFc was also generated through a mutation of the processing site to further establish that the results with the SC rFVIIIFc were not due to the divalent cation removal and re-introduction during purification of SC rFVIIIFc from rFVIIIFc. Although SDS-PAGE analysis showed this SC rFVIIIFc R1645A/R1648A predominantly contains the single chain species, this protein was also found to contain alternatively processed species due to other truncations commonly found in recombinant FVIII proteins (e.g. cleavage at E720, Y729, D1658, see Figure 1 and Figures S1, S2 in File S1), therefore this comparator was a mixture of single chain and dual chain rFVIIIFc species. The functional activity of SC rFVIIIFc R1645A/R1648A was similar to the SC rFVIIIFc in the assays in the context of the Xase complex and in the chromogenic activity assay, thus indicating that the SC rFVIIIFc activity was not adversely affected by dissociation/re-association or other aspects of the purification processes. The aPTT activity of the SC rFVIIIFc R1645A/R1648A in the presence of VWF was found to be intermediate to that of SC rFVIIIFc and rFVIIIFc in preliminary studies (data not shown), consistent with the presence of fully processed rFVIIIFc in the content, and therefore this test article was omitted from later analyses.

Given the comparable affinities of SC rFVIIIFc, rFVIIIFc and BDD rFVIII for VWF observed in SPR (Table 3 and previously described [10]), the discrepancy in the specific activity of SC rFVIIIFc by aPTT in the presence of VWF do not appear to be attributable to affinity for VWF. We therefore explored the alternative possibility that the lack of proteolytic processing in SC rFVIIIFc could significantly alter the kinetics of release from VWF following activation by thrombin (Figure 4), thereby limiting the availability of activated SC rFVIIIFc for Xase complex formation. Upon activation by either thrombin or FXa, FVIII is ordinarily cleaved at R372, R740 and R1689, resulting in an unstable, metal ion-dependent A1/A2/A3-C1-C2 heterotrimer that possesses cofactor activity [26],[27]. In the case of fully processed FVIII (*i.e.,* separate HC and LC), cleavage after R1689 during activation liberates the a3 acidic peptide from the N-terminus of the LC, resulting in release of the cofactor from VWF, and this step is required for Xase complex assembly and thus contributes to the overall activity of the cofactor. In the case of the SC rFVIIIFc, which has not been proteolytically processed at R1648, thrombin cleavage after R1689, should it occur prior to cleavage after R740, may cause delayed dissociation of the a3 peptide due to sustained covalent linkage to the heavy chain, resulting in delayed release of activated SC rFVIIIFc from VWF, delayed assembly of the Xase complex and lower apparent activity in the one-stage clotting assay. This impairment of thrombin-mediated release from VWF was demonstrated by the increased thrombin EC_50_ values (Figure 4), and is consistent with the reduction in specific activity observed in the aPTT assay format in which VWF was present. Upon complete cleavage, activated SC rFVIIIFc is structurally and functionally equivalent to activated rFVIIIFc, confirmed by the results of the two-stage chromogenic assay and biochemical characterization performed in the context of Xase complex (Tables 1 and 2, Figure 3).

In the thrombin generation assay, SC rFVIIIFc demonstrated moderately reduced peak thrombin and ETP, as well as prolonged time to peak. This is a manifestation of delayed release of SC rFVIIIFc from VWF in a dynamic system with limiting thrombin and FXa in the initiation phase of coagulation. In contrast, rFVIIIFc and fully processed rFVIIIFc generated identical thrombin generation profiles to WHO FVIII concentrate standard at all concentrations (Figure 2).

SC rFVIIIFc might afford enhanced stability in vivo and contribute to improved half-life and efficacy. However, the pharmacokinetics of the SC rFVIIIFc were examined in hemophilia A mice and found to be similar to those of rFVIIIFc (Figure 6 and Table 4). Both SC rFVIIIFc and rFVIIIFc have comparable prophylactic efficacy in protecting HemA mice from the venous injury, which mimics the spontaneous capillary bleeds in severe hemophilia A patients. In this model, the comparable dose responses in rebleed rates also suggest that the quality and stability of the clot formed by SC rFVIIIFc and rFVIIIFc are indistinguishable (Figure 5). Thus, no enhanced stability from the SC rFVIIIFc is detectable in vivo. Furthermore, the delayed activation of SC rFVIIIFc and initiation of clot formation in the presence of VWF observed in vitro under certain conditions in TGA and one-stage clotting assays appears to have no significant impact on its *in vivo* efficacy and PK profile, which may depend less on the initial rate of FVIIIa formation and more on overall clot development and stability.

In conclusion, we characterized a novel single chain isoform of rFVIIIFc that is fully functional in vivo despite reduced activity in one-stage clotting assay in the presence of VWF, which results from delayed thrombin-mediated release from VWF. Our results indicate that the in vivo activity of SC rFVIIIFc is not enhanced but comparable to rFVIIIFc.

## Supporting Information

File S1 **Figures S1–S5 and Table S1.** Figure S1. Mass spectra of 6 kDa LC N-terminus peptide fragment of FVIII variants after thrombin digestion and LC-MS (TOF) analysis. (a) rFVIIIFc (b) BDD rFVIII (c) SC rFVIIIFc (d) SC rFVIIIFc R1645A/R1648A. Major digestion products are indicated. Figure S2. Deconvoluted spectra of A2 fragment of FVIII variants after thrombin digestion and LC-MS (TOF) analysis. (a) rFVIIIFc (b) BDD rFVIII (c) SC rFVIIIFc (d) SC rFVIIIFc R1645A/R1648A. Major digestion products are indicated. Figure S3. Representative SPR sensorgrams of interactions between FVIII variants and VWF. One representative graph is displayed for (A) rFVIIIFc and (B) SC rFVIIIFc, respectively. Black indicates the binding curve and red indicates the best fit to a 1∶1 interaction model. Figure S4. Thrombin-mediated release of activated FVIII variants from VWF at 25°C. (A) Single reference subtracted sensorgrams. (B) Double reference subtracted sensorgrams for the phase corresponding to thrombin application. (C) Thrombin-mediated release rate as a function of time. (D) Peak thrombin-mediated release rate as a function of thrombin concentration. Figure S5. Thrombin-mediated release of activated FVIII variants from VWF at 37°C. (A) Single reference subtracted sensorgrams. (B) Double reference subtracted sensorgrams for the phase corresponding to thrombin application. (C) Thrombin-mediated release rate as a function of time. (D) Peak thrombin-mediated release rate as a function of thrombin concentration. Table S1. Summary of results from thrombin map by LC-MS (TOF) analysis. For SC rFVIIIFc R1645A/R1648A, mass analysis confirmed the presence of the R1645A/R1648A mutations.
(DOCX)
