# Supplementary material for: A Single Chain Variant of Factor VIII Fc Fusion Protein Retains Normal In Vivo Efficacy but Exhibits Altered In Vitro Activity
Source: PLoS One. 2014 Nov 21;9(11):e113600. doi: 10.1371/journal.pone.0113600 (PMC4240654; doi:10.1371/journal.pone.0113600)

**Material and Methods:**

*Purification of SC rFVIIIFc*

SC rFVIIIFc was purified from rFVIIIFc using the following steps: first the FVIII HC of processed rFVIIIFc was dissociated from FVIII LC-Fc fusion protein via EDTA mediated chelation of divalent cations while the HC and LC of SC rFVIIIFc remained covalently attached. The SC rFVIIIFc, together with the dissociated rFVIIIFc heavy chain, was then separated from the dissociated rFVIIIFc light chain-Fc fusion using anion exchange chromatography. A buffer containing divalent cations (16 mM Ca^2+^ and 2 µM Cu^2+^) was used to restore the non-covalent HC-LC interaction in SC rFVIIIFc. SC rFVIIIFc was then separated from the dissociated FVIII HC using Fc affinity chromatography (soluble human FcRn conjugated to Sepharose beads). After concentration with a Pellicon XL TFF Cassette (Millipore, Billerica, MA), followed by a size exclusion chromatography to remove aggregated species, SC rFVIIIFc was concentrated by centrifugal ultrafiltration.

*Surface plasmon resonance analysis of von Willebrand Factor binding*

All analyses were performed on a Biacore T100 instrument (GE Healthcare, Piscataway, NJ, USA) at 25°C. Approximately 500 resonance units of purified FVIII-free plasma-derived human von Willebrand Factor (hVWF; Haematologic Technologies, Inc, Essex Junction, VT, USA) were immobilized at each of three flow cells of a CM5 biosensor chip (GE Healthcare, Piscataway, NJ, USA) by standard amine coupling at pH 5, and residual-activated sites were subsequently blocked with ethanolamine. rFVIIIFc and SC rFVIIIFc analyte samples were applied sequentially at 25 µL/min in single-cycle kinetics mode at concentrations of 0.13, 0.32, 0.8, 2.0, and 5.0 nM, each for 240 seconds followed by a terminal dissociation phase of 1200 seconds. Binding and dissociation steps were performed in a buffer that consisted of 6.7 mM L-histidine, 5.3 mM CaCl_2_, 205 mM NaCl, 1.3% sucrose, and 0.013% polysorbate 20, pH 7.0. The hVWF coupled chip surface was regenerated between cycles with a solution of 0.6 M NaCl and 0.35 M CaCl_2_. Raw data were processed by double reference subtraction, and kinetic parameters were derived from a 1:1 binding model that yielded values for χ^2^/R_max_ of 0.02 or less.

*SPR analysis of thrombin-mediated release of activated FVIII variants from VWF*

Thrombin-mediated FVIII release assays were performed with a Biacore T100 instrument and consisted of four steps (Figure 4A). In step 1, approximately 2500 RUs of human VWF were covalently immobilized at 25°C on each of three flow cells of a CM5 biosensor chip (GE Healthcare) by standard amine coupling at pH 5, and residual activated sites were blocked with ethanolamine. All subsequent steps were performed at 37°C at a flow rate of 25 μL/min. In step 2, SC rFVIIIFc, rFVIIIFc or BDD rFVIII at 50 nM in Running Buffer was applied for 180 s to allow association with immobilized VWF. In step 3, the chip was infused with Running Buffer for 115 s to enable dissociation of FVIII variants such that equal-molar amounts of each were captured by the end of the dissociation phase (approximately 550 RU of rFVIIIFc and SC FVIIIFc, and approximately 425 RU of BDD rFVIII). In step 4, plasma-derived human α-thrombin (Haematologic Technologies, specific activity 5380 U/mg) (0.005 to 20 U/mL, corresponding to 0.025 to 100 nM) or Running Buffer devoid of thrombin were applied for 120 s, followed by infusion of Running Buffer for an additional 120 s. After the initial coupling of VWF to the chip (step 1), steps 2-4 were repeated in serial cycles with different thrombin concentrations. Residual FVIII was removed between cycles by the application of Regeneration Buffer for 60 s. Each cycle resulted in three single reference subtracted sensorgrams in which the signal from the blank flow cell was subtracted from those from the flow cells in which VWF had been immobilized. Double reference subtracted sensorgrams were then generated for the phase corresponding to thrombin application by subtracting the signal resulting from the Running Buffer blank injection. Maximum release rates (expressed in units of RU/s) for each FVIII variant at each thrombin concentration were derived from the negative first derivative of double reference subtracted sensorgrams. The concentration of thrombin corresponding to the half-maximal release rate (EC_50_) for each FVIII variant was determined by non-linear least squares regression using the following two variable hyperbolic function and solving for EC_50_:

$y=$Rate_max_**x*/(EC_50_ + *x*)

where *y* is the maximum release rate (in units of RU/s) as a function of thrombin concentration *x* (in units of U/mL), and Rate_max_ is the theoretical maximum release rate (in units of RU/s) at infinite thrombin concentration.

**Figures:**

**Figure S1:** Mass spectra of 6 kDa LC N-terminus peptide fragment of (a) rFVIIIFc (b) BDD rFVIII (c) SC rFVIIIFc (d) SC rFVIIIFc R1645A/R1648A after thrombin digestion and LC-MS (TOF) analysis.

(a)

x10 ^3^

0

0.25

0.5

0.75

1

1.25

1.5

1.75

2

2.25

1276.2978

1701.3918

819.0169

1021.4398

1446.6290

Mass-to-Charge (m/z)

800

900

1000

1100

1200

1300

1400

1500

1600

1700

1800

Processed

a3 E^1649^-R^1689^

4+

3+

**rFVIIIFc**

Relative signal

0

0.2

0.4

0.6

0.8

1

1130.5207

1356.6261

819.0168

1288.5760

969.3034

1695.5327

1546.0931

Mass-to-Charge (m/z)

800

900

1000

1100

1200

1300

1400

1500

1600

1700

1800

Single chain

Linker-a3 S^741^-R^1689^

6+

5+

4+

Truncated

Δa3 D^1658^-R^1689^

1018.9

4+

1358.4

3+

x10 ^3^

Relative signal

**rFVIIIFc**

(b)

0

0.5

1

1.5

2

2.5

3

3.5

4

4.5

1276.5475

1023.2699

1701.3936

Mass-to-Charge (m/z)

900

1000

1100

1200

1300

1400

1500

1600

1700

1800

1900

3+

4+

Processed

a3 E^1649^-R^1689^

**BDD rFVIII**

Relative signal

x10 ^2^

x10 ^2^

0

0.5

1

1.5

2

2.5

3

3.5

4

4.5

1358.2096

1019.1584

1130.6914

Mass-to-Charge (m/z)

950

1000

1050

1100

1150

1200

1250

1300

1350

1400

1450

1356.4

6+

5+

3+

4+

Truncated

Δa3 D^1658^-R^1689^

Single chain

Linker-a3 S^741^-R^1689^

**BDD rFVIII**

Relative signal

(c)

x10 ^3^

0

0.5

1

1.5

2

2.5

3

3.5

1130.6908

1356.4262

1288.5801

969.1655

1695.5260

1546.2962

819.0224

Mass-to-Charge (m/z)

800

900

1000

1100

1200

1300

1400

1500

1600

1700

6+

5+

4+

Relative signal

Single chain

Linker-a3 S^741^-R^1689^

**SC rFVIIIFc**

(d)

x10 ^3^

0

0.5

1

1.5

2

2.5

3

819.0160

1322.5955

1023.5196

682.6824

1102.3293

1512.0622

1652.9941

945.1450

Mass-to-Charge (m/z)

700

800

900

1000

1100

1200

1300

1400

1500

1600

1700

4+

5+

6+

7+

Single chain

Linker-a3, S^741^-R^1689^

with mutations

R^1645^, R^1648^―A

**SC rFVIIIFc**

**R1645A/R1648A**

Relative signal

x10 ^2^

0

0.2

0.4

0.6

0.8

1

1.2

1.4

1.6

1023.5208

1358.5409

Mass-to-Charge (m/z)

900

1000

1100

1200

1300

1400

1500

1600

1700

1800

1900

3+

4+

1018.9

Truncated

Δa3 D^1658^-R^1689^

**SC rFVIIIFc R1645A/R1648A**

Relative signal

**Figure S2:** Deconvoluted spectra of A2 fragment of (a) rFVIIIFc (b) BDD rFVIII (c) SC rFVIIIFc (d) SC rFVIIIFc R1645A/R1648A after thrombin digestion and LC-MS (TOF) analysis.

(a)

x10 ^5^

0

0.2

0.4

0.6

0.8

1

1.2

1.4

42725.24

41488.76

40364.61

45927.82

44226.74

45195.37

Deconvoluted Mass (amu)

40500

41000

41500

42000

42500

43000

43500

44000

44500

45000

45500

46000

**A2**

**^373^S-Y^729^**

**^373^S-E^720^**

Relative signal

**^373^S-R^740^**

**rFVIIIFc**

(b)

x10 ^4^

0

1

2

3

4

5

6

7

42725.16

40364.63

41489.47

45193.42

44297.68

43676.58

Deconvoluted Mass (amu)

40500

41000

41500

42000

42500

43000

43500

44000

44500

45000

**BDD rFVIII**

**A2**

**^373^S-R^740^**

**^373^S-Y^729^**

**^373^S-E^720^**

Relative signal

(c)

x10 ^5^

0

0.25

0.5

0.75

1

1.25

1.5

1.75

2

2.25

42725.30

41792.84

44315.13

45102.63

45924.82

40978.59

Deconvoluted Mass (amu)

40500

41000

41500

42000

42500

43000

43500

44000

44500

45000

45500

46000

**SC rFVIIIFc**

**A2**

**^373^S-R^740^**

Relative signal

(d)

0

0.2

0.4

0.6

0.8

1

1.2

1.4

1.6

1.8

42725.22

41488.99

40364.00

44437.63

45037.63

43672.86

Deconvoluted Mass (amu)

40500

41000

41500

42000

42500

43000

43500

44000

44500

45000

**SC rFVIIIFc**

**R1645A/R1648A**

**A2**

**^373^S-R^740^**

**^373^S-Y^729^**

**^373^S-E^720^**

Relative signal

x10 ^5^

**Table S1: Summary of results from thrombin map by LC-MS TOP analysis.** For SC rFVIIIFc 1645A/1648A, mass analysis confirmed the presence of the R1645A/R1648A mutations.

| **Sample Analyzed** | **Regions confirmed** | **Comments** |
| --- | --- | --- |
| rFVIIIFc | A1, A2, a3-related peptide, LC-Fc, Fc | Observed processed, single chain, and truncated a3  Observed full length and truncated A2 |
| BDD rFVIII | A1, A2, a3-related peptide, LC | Observed processed, single chain, and truncated a3  Observed full length and truncated A2 |
| SC rFVIIIFc | A1, A2, a3-related peptide, LC-Fc, Fc | Observed single chain a3 only  Observed full length A2 only |
| SC rFVIIIFc R1645A/R1648A | A1, A2, a3-related peptide, LC-Fc, Fc | Observed single chain and truncated a3  Observed full length and truncated A2 |

**Figure S3: Representative SPR sensorgrams of interactions between (A) rFVIIIFc or (B) SC rFVIIIFc and VWF.** Experiments were performed in single-cycle kinetics mode, and data were fit to a simple 1:1 binding model. A total of 6 binding experiments were performed for each interaction to generate data presented in Table 3. One representative graph is displayed here. Black indicates the binding curve and red indicates the best fit to a 1:1 interaction model.

**
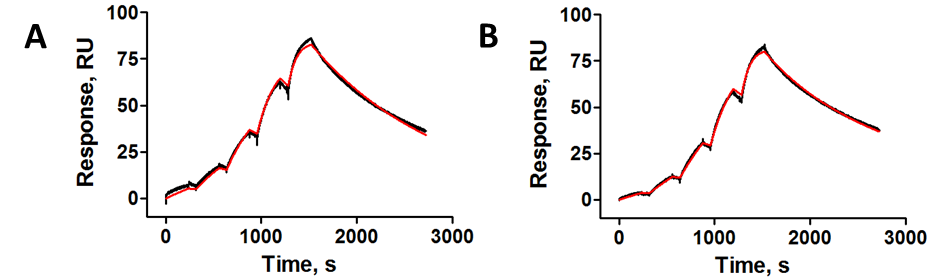
**

**Figure S4: Thrombin-mediated release of activated FVIII variants from VWF at 25°C.** (A) Single reference subtracted sensorgrams. (B) Double reference subtracted sensorgrams for the phase corresponding to thrombin application. (C) Thrombin-mediated release rate as a function of time. (D) Peak thrombin-mediated release rate as a function of thrombin concentration.


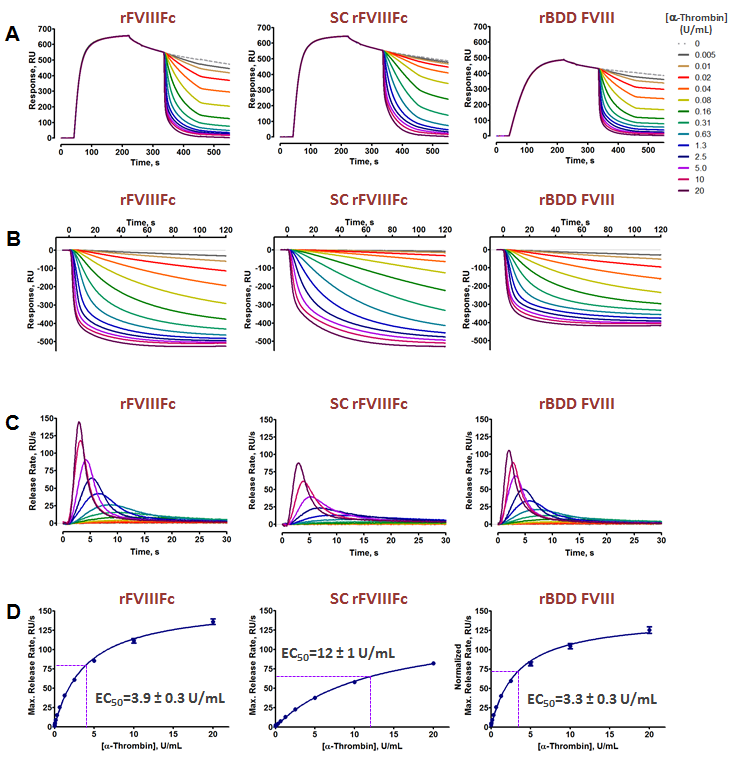


**Figure S5: Thrombin-mediated release of activated FVIII variants from VWF at 37°C.** (A) Single reference subtracted sensorgrams. (B) Double reference subtracted sensorgrams for the phase corresponding to thrombin application. (C) Thrombin-mediated release rate as a function of time. (D) Peak thrombin-mediated release rate as a function of thrombin concentration.


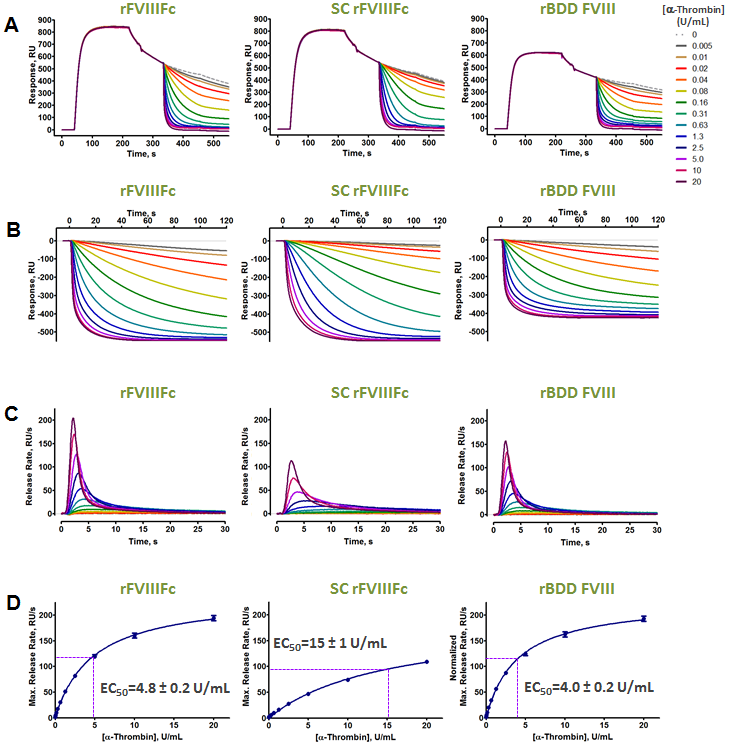

Supplement: File S1 — Figures S1–S5 and Table S1. Figure S1. Mass spectra of 6 kDa LC N-terminus peptide fragment of FVIII variants after thrombin digestion and LC-MS (TOF) analysis. (a) rFVIIIFc (b) BDD rFVIII (c) SC rFVIIIFc (d) SC rFVIIIFc R1645A/R1648A. Major digestion products are indicated. Figure S2. Deconvoluted spectra of A2 fragment of FVIII variants after thrombin digestion and LC-MS (TOF) analysis. (a) rFVIIIFc (b) BDD rFVIII (c) SC rFVIIIFc (d) SC rFVIIIFc R1645A/R1648A. Major digestion products are indicated. Figure S3. Representative SPR sensorgrams of interactions between FVIII variants and VWF. One representative graph is displayed for (A) rFVIIIFc and (B) SC rFVIIIFc, respectively. Black indicates the binding curve and red indicates the best fit to a 1∶1 interaction model. Figure S4. Thrombin-mediated release of activated FVIII variants from VWF at 25°C. (A) Single reference subtracted sensorgrams. (B) Double reference subtracted sensorgrams for the phase corresponding to thrombin application. (C) Thrombin-mediated release rate as a function of time. (D) Peak thrombin-mediated release rate as a function of thrombin concentration. Figure S5. Thrombin-mediated release of activated FVIII variants from VWF at 37°C. (A) Single reference subtracted sensorgrams. (B) Double reference subtracted sensorgrams for the phase corresponding to thrombin application. (C) Thrombin-mediated release rate as a function of time. (D) Peak thrombin-mediated release rate as a function of thrombin concentration. Table S1. Summary of results from thrombin map by LC-MS (TOF) analysis. For SC rFVIIIFc R1645A/R1648A, mass analysis confirmed the presence of the R1645A/R1648A mutations. (DOCX) [file pone.0113600.s001.docx]
